# 智能聚合物基材料富集磷酸化肽和糖肽的研究进展

**DOI:** 10.3724/SP.J.1123.2020.05036

**Published:** 2021-01-08

**Authors:** Xintong ZHENG, Xue WANG, Fusheng ZHANG, Xuyang ZHANG, Yanyan ZHAO, Guangyan QING

**Affiliations:** 1.大连医科大学药学院, 辽宁 大连 116044; 1. Pharmacy College, Dalian Medical University, Dalian 116044, China; 2.中国科学院分离分析化学重点实验室, 中国科学院大连化学物理研究所, 辽宁 大连 116023; 2. Key Laboratory of Separation Science for Analytical Chemistry, Dalian Institute of Chemical Physics, Chinese Academy of Sciences, Dalian 116023, China

**Keywords:** 蛋白质组学, 富集, 翻译后修饰, 智能聚合物, 磷酸化肽, 糖肽, 综述, proteomics, enrichment, post-translational modification (PTM), smart polymer, phosphorylated peptides, glycopeptides, review

## Abstract

翻译后修饰是蛋白质组学研究的前沿和重点,它不仅调节着蛋白质的折叠、状态、活性、定位以及蛋白质间的相互作用,也能帮助科学家更全面地了解生物体的生命过程,为疾病的预测、诊断和治疗提供更加强大的支撑和依据。翻译后修饰产物(例如磷酸化肽和糖肽)丰度很低,且存在着强烈的背景干扰,很难直接用质谱进行分析,因此迫切需要开发高效的富集材料和技术来选择性富集翻译后修饰产物。近年来,智能聚合物基材料通过外部物理、化学或生物刺激可逆地改变其结构和功能,实现对磷酸化肽和糖肽高度可控的吸附和脱附,进而衍生开发出一系列新颖的富集方法,极大地吸引研究者们的兴趣。一方面,智能聚合物基材料的响应变化包括材料疏水性的增加或减少、形状和形貌的改变、表面电荷的重新分布以及亲和配体的暴露或隐藏等特性。这些特性使得目标物和智能聚合物基材料之间的亲和力可以通过简单改变外部条件(如温度、pH值、溶剂极性和生物分子等)实现更可控和更智能的精细调节。另一方面,智能聚合物基材料为集成功能模块提供了便捷的可扩展平台,例如特定的识别组件,显著提高了目标物质的分离选择性。智能聚合物基材料在分离方面展现出巨大的潜力,这为蛋白质翻译后修饰产物的分析和研究带来了希望。围绕上述主题,该文依据Web of Science近20年来近50篇代表性文献,概述了智能聚合物基材料在磷酸化肽和糖肽分离及富集中的发展方向。

## Abstract (en)

Protein post-translational modification (PTM) is at the forefront of focus of proteomics research. It not only regulates protein folding, state, activity, localization, and protein interactions, but also helps scientists understand the biological processes of organisms more comprehensively, providing stronger support and basis for the prediction, diagnosis, and treatment of diseases. In living organisms, there are more than 300 types of PTMs of proteins and their modification processes are dynamic. At the same time, protein modifications do not exist in isolation. The occurrence of the same physiological or pathological process requires the joint action of various modified proteins, which affect and coordinate with each other. Owing to the low abundance of PTM products (e. g., phosphorylated peptides or glycopeptides) and the presence of strong background interference, it is difficult to analyze them directly through mass spectrometry. Therefore, the development efficient materials and techniques for the selective enrichment of PTM peptides is urgently needed. Conventional separation methods have partially solved the challenges involved in the enrichment of glycopeptides and phosphorylated peptides; however, there are some inevitable issues, such as the excessive binding force of metal ions (e. g., Fe^3+^and Ti^4+^) toward multiple phosphorylated peptides, resulting in difficulty in elution and identification through mass spectrometry. In addition, owing to the insufficient binding affinity of materials toward glycopeptides, most glycopeptides that have been identified at present are of the sialic acid type, and a large number of neutral glycans, for instance, *O*-link glycopeptides and high mannose-type glycans are difficult to enrich and identify.

The emergence of smart polymers provides a new avenue for the development of PTM-enriched materials. Several studies have reported that smart polymers can reversibly change their structure and function through external physical, chemical, or biological stimulation, to achieve highly controllable adsorption and desorption of phosphorylated peptides and glycopeptides. Based on this strategy, a series of novel enrichment materials and methods have been developed, which have greatly attracted the interest of researchers. On the one hand, the response changes of smart polymers include the increase or decrease of hydrophobicity, the change of shape and morphology, the redistribution of surface charge, the exposure or hiding of affinity ligands, etc. Changes in these properties can be achieved by simply changing external conditions such as temperature, pH, solvent polarity, and biomolecules. These properties, in turn, enable the fine-tuning of the affinity between the target and the smart polymers. Furthermore, the affinity can provide an additional driving force, which can significantly improve biological separation.

On the other hand, smart polymers provide a series of convenient and expandable platforms for integrating various functional modules, such as specific recognition components, which will facilitate the development of novel enrichment materials for protein methylation, acetylation, and ubiquitination. Smart polymer materials show great potential in the field of separation, which is promising for the analysis and research of protein PTMs. This review summarizes the research progress of smart polymer materials for the separation and enrichment of phosphorylated peptides and glycopeptides according to nearly 50 representative articles from the Web of Science in the past two decades.

自从人类基因组计划完成后,基因组学、蛋白质组学、糖组学、酶组学、代谢组学等^[1,2,3,4]^各种形式的“组学”研究逐渐蓬勃发展起来。蛋白质组学从整体的角度分析生物体细胞内动态的蛋白质组成,以及表达水平与翻译后修饰状态,进而了解蛋白质功能与生命体活动之间的规律;其中翻译后修饰使蛋白质的结构更为复杂,功能更为完善,调节更为精细,作用更为专一。蛋白质翻译后修饰是一个非常复杂的过程,目前在真核生物中有多达300多种的修饰种类^[5]^,蛋白质的磷酸化和糖基化是最基础、最普遍,也是最重要的翻译后修饰方式,在信号转导、免疫应答和细胞分化等方面起着关键的调控作用^[6]^。同时肿瘤的发生、发展和转移与异常的蛋白糖基化和磷酸化关系密切^[7,8]^,其具有十分重要的生物学意义和临床应用价值。

然而,在复杂的生物样品体系中,磷酸化蛋白和糖蛋白的含量极其微量,并且翻译后修饰动态可变性大;此外,糖肽、磷酸化肽在质谱中离子化效率低,存在强烈的背景干扰等问题,因而很难用质谱对其进行直接分析,因此迫切需要对样品进行高效的分离和富集,才能获得较高选择性和较广类型覆盖范围的磷酸化肽和糖肽。糖肽的富集方法主要包括凝集素亲和色谱法^[9]^、肼化学法^[10]^、苯硼酸亲和色谱法^[11]^、亲水色谱法^[12]^等。磷酸化肽的富集方法主要有固定金属离子亲和色谱法^[13]^和金属氧化物亲和色谱法^[14]^等,而这些传统的分离方法部分解决了糖肽和磷酸化肽富集的难题,但是也有一些不可避免的问题,例如基于金属离子对多磷酸化肽的结合力过大,导致其难以洗脱和被质谱鉴定。此外,材料对糖肽的捕获效率不足,目前被鉴定出的糖肽大多为唾液酸型,大量的中性糖、*O*-糖肽难以被富集和鉴定。

智能聚合物基材料是一类新涌现的材料,基于自身的智能属性,即其物理和化学性质(例如高分子构象、表面形貌、浸润性、黏弹性、吸附性能等)对温度、pH值、溶剂、光、电场或生物分子等外界刺激,具有灵敏、快速、显著和可逆的响应性,已被广泛应用于纳米材料、生物、医学等多个领域^[15,16,17]^。借助聚合物链可控的构象转变及由此产生的开-关效应,智能聚合物基材料在可控药物递送和释放、组织工程等方面展现出很好的应用前景,但其在分离分析领域的潜力还未引起人们足够的关注。新型材料设计理念的引入,有可能开发出对生物、医药和环境中特殊生物分子灵敏和高效的分离技术与检测方法。对于翻译后修饰蛋白质组学而言,更需要融入先进的方法和技术,以显著提升富集的效率和对修饰肽链的覆盖率,从而弥补现有富集材料在富集效率和覆盖率方面的不足。近年来,智能聚合物基材料在磷酸化肽和糖肽分离及富集领域取得了一些进展(见表1),为此我们撰写本篇综述,总结智能材料在该领域中的应用现状,分析其优势、问题,以迎接未来的挑战。

**表 1:** 用于富集翻译后修饰产物的代表性智能聚合物基材料

Material type	Target	Real biological sample	Stimulus	Binding mode	Ref.
PNIPAM-co-	phosphorylated peptides	HeLa S3 cell lysate	pH/temperature/	hydrogen bonds	[18]
ATBA_0.2_@SiO_2_			solvent polarity		
PNIPAM-co-	phosphorylated peptides and	HeLa cell lysate	pH	hydrogen bonds	[19]
ATBA_0.2_@SiO_2_	sialylated glycopeptides				
Fe_3_O_4_/PDA/PAMA-Arg	phosphorylated peptides	rat brain lysate	solvent polarity	hydrogen bonds	[20]
Fe_3_O_4_@PGMA-guanidyl	phosphorylated peptides	tryptic digest of	solvent polarity	-	[21]
		nonfat milk			
TMIPs	phosphorylated peptides	-	temperature	size of the imprinting	[22]
				cavities	
Poly-(AA-co-hydrazide)	glycopeptides	mouse brain lysate	pH	covalent bonds	[23]
PNIPAM-TP polymer	glycoprotein	HeLa cell lysate	temperature	covalent bonds	[24]
b-PMMA spheres	glycoprotein	egg white	pH/temperature	covalent bonds	[25]
Poly(Pro-Glu)@SiO_2_	glycopeptides	HeLa cell lysate	pH	hydrogen bonds	[26,27]
Fe_3_O_4_@PMAH	glycopeptides	colorectal cancer	-	covalent bonds	[28]
		patient serum			

PNIPAM: poly(*N*-isopropyl-acrylamide); ATBA: 4-(3-acryloyl-thioureido)-benzoic acid; PDA: polydopamine; PAMA: poly(2-aminoethyl methacrylate hydrochloride); PGMA: poly(glycidyl methacrylate); TMIPs: thermosensitive molecularly imprinted polymers; AA: acrylic acid; TP: triarylphosphine; b: boronic acid group-bearing; PMMA: polymethyl methacrylate; Pro-Glu: proline-glutamic; PMAH: poly(methacrylic hydrazide); -: no data.

## 1 智能聚合物基磷酸化肽富集材料

蛋白质磷酸化是指蛋白质分子中的丝氨酸、苏氨酸或酪氨酸残基在蛋白激酶的作用下,从三磷酸腺苷(ATP)分子上获取磷酸基团而发生磷酸化的过程。磷酸化是大自然中最广泛的翻译后修饰形式,据统计,在哺乳动物细胞中,30%以上的蛋白质会发生不同程度的磷酸化。同时,蛋白质磷酸化又是一个可逆过程,在磷酸酯酶的作用下,磷酸化的氨基酸残基又可以发生去磷酸化过程。蛋白质磷酸化尽管修饰形式和基团较为简单,但是却在生命活动中扮演着极为重要的角色,例如调节大量细胞活动如信号转导、细胞分裂、分化和代谢等^[29,30,31,32]^。此外,在病理意义上,异常磷酸化更是与许多人类疾病密切相关,如阿尔茨海默病(AD)^[33]^和癌症。阿尔茨海默病的病理特征之一就是因微管结合蛋白Tau的异常蛋白质磷酸化所产生的神经原纤维缠结,尤其在多点磷酸化微管蛋白结合区域,可以累积成不溶性配对螺旋细丝,产生许多神经原纤维缠结。然而,值得注意的是磷酸化肽的天然丰度很低,离子化效率也较低,同时样品中还存在着大量具有强烈背景干扰的高丰度非修饰肽段^[34,35]^。因此,在进行质谱分析前,首要任务是从复杂的生物样品中高效专一地选择性富集目标分析物,提高质谱分析样品中磷酸化肽的浓度,并消除样品基质中非修饰肽、盐等各类干扰成分。

多位点磷酸化是细胞中最重要且常见的修饰方式。然而由于富集材料的限制,从复杂的生物样品中捕获低丰度多磷酸化肽的难度很大,因此对于多磷酸化肽的研究远远落后于单磷酸化肽。这主要是因为基于多重金属螯合的主流方法成倍地增加了基质金属与多磷酸化肽的亲和力,然而这种螯合作用过于强烈,不易调控,使得结合的多磷酸化肽难以从材料表面解离下来。除了洗脱难题,大多数富集材料对酸性干扰肽也存在严重的非特异性吸附,大大降低了材料的富集选择性。Qing等^[18]^针对上述多磷酸化肽的选择性富集难题设计了一种基于氢键的智能共聚物。该共聚物包含一个高效的磷酸识别单体-对羧基苯基硫脲(ATBA),以及一个柔性聚*N*-异丙基丙烯酰胺(PNIPAM)网络,他们将该聚合物与多孔硅胶基质进行复合,制备了一种新型的多磷酸化肽富集材料。该材料的特点包括材料与磷酸化肽通过动态可逆的多重氢键进行结合;对多磷酸化肽高度可控的吸附行为可以通过溶剂极性、pH和温度等环境参数进行动态调控;对磷酸化肽具有超高的吸附容量和富集回收率;对稀有的酪氨酸磷酸化肽展现出独特的亲和力。正是基于上述特点,该材料成功地从人宫颈癌细胞裂解液中富集得到了1257条磷酸化肽(其中71%为多磷酸化肽),并且通过质谱鉴定发现了大量之前从未被报道的磷酸化新位点。在这些新位点中,稀有的苏氨酸和酪氨酸磷酸化位点的比例明显提高。智能聚合物基材料这一优异的富集效果,揭示了其在多磷酸化肽富集中的独特优势,为丰富和推进磷酸化蛋白组学研究提供了一种新型、强有力的富集工具。

基于相似的策略,Lu等^[19]^进一步开发了对磷酸化肽和唾液酸糖肽具有同步富集能力的智能聚合物基材料。他们发现对羧基苯基硫脲功能单体既可以通过氢键作用实现对磷酸化位点的精确识别,也可以选择性地结合唾液酸。聚合物链初始时处于紧缩的状态,在结合多磷酸化肽或唾液酸糖肽之后发生了从紧缩向完全舒张的转变,显著提升了对磷酸化肽和唾液酸糖肽这些肽链的吸附(见图1)。

**图 1:**
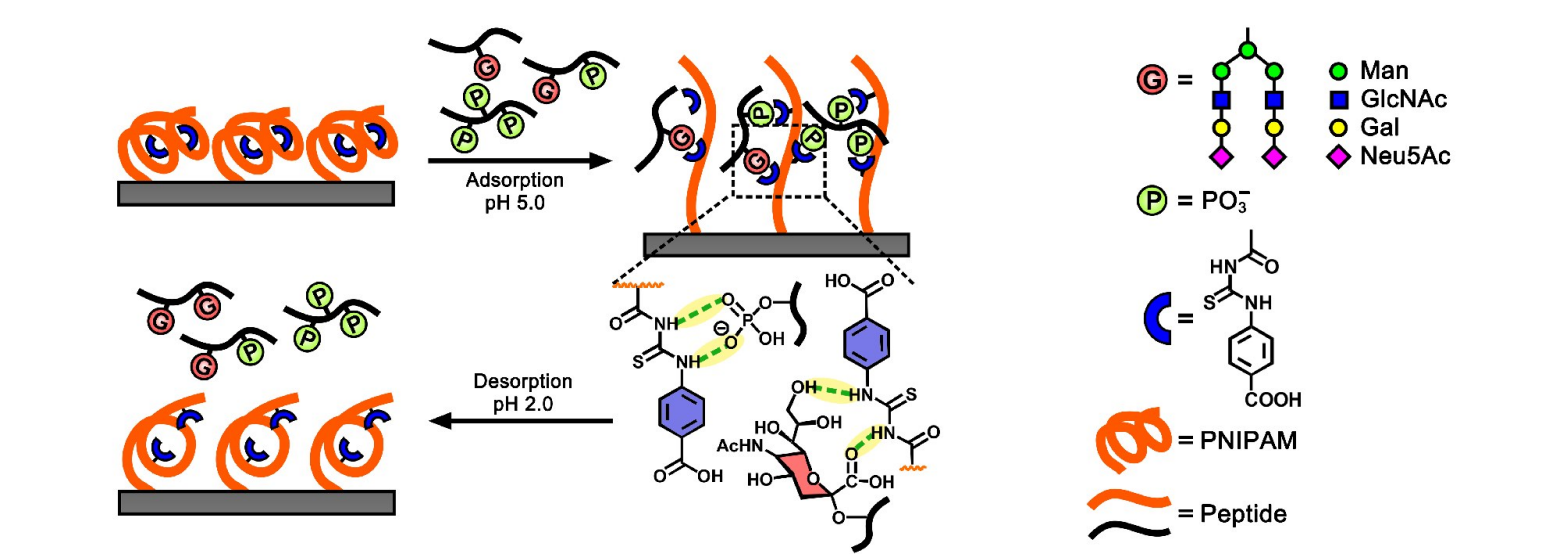
智能聚合物基材料对多磷酸化肽和唾液酸型糖肽的可控吸附和脱附行为^[19]^

进一步提高溶液的酸性后,强质子的环境破坏了硫脲单元与磷酸化位点或唾液酸化位点之间的氢键作用,使得捕获的多磷酸化肽、唾液酸糖肽又从材料表面解离下来。该智能共聚物具有很高的唾液酸糖肽吸附容量(370 mg/g)和高回收率,以及高的富集选择性,可以从50 μg人宫颈癌细胞HeLa细胞裂解液中富集得到糖肽和磷酸化肽的占比为79%,从631个磷酸化肽中产生721个独特的磷酸化位点,从120个糖肽中产生125个独特的糖基化位点,这将为翻译后修饰富集材料的开发开辟出一条新途径。

表面引发原子转移自由基聚合技术由于接枝密度高、聚合物链长度可控等优势,被广泛用于在不同的基底接枝不同的聚合物。Qin等^[36]^利用这种技术开发了一种新型的毛细管柱,并成功地将其与LC-MS系统联合,应用于自动在线磷酸化肽的富集和鉴定。这种聚合技术开发的新型毛细管柱从HepG2细胞裂解液中鉴定出475条磷酸化肽以及662个磷酸化位点,远高于利用TiO_2_在线富集的39条磷酸化肽。更重要的是,这种聚合技术可以与各种功能化单体有效兼容,并且不需要严苛的实验条件,这为富集材料的开发提供了一种新思路。

2018年,Luo等^[20]^通过原子转移自由基聚合技术合成Fe_3_O_4_/聚多巴胺/聚(2-氨基乙基甲基丙烯酸酯盐酸盐)-精氨酸(Fe_3_O_4_/PDA/PAMA-Arg)纳米复合微球。核心的PAMA-Arg聚合物刷代替了传统的金属离子螯合体系,有效避免了材料在上样和洗脱过程中金属离子的流失。同时聚合物刷舒展的三维空间结构,显著促进了精氨酸胍基与磷酸化肽间的相互作用,实现了对磷酸化肽的高效富集。此外,Fe_3_O_4_良好的磁性能简化了分离过程,避免了烦琐的离心操作,有助于磷酸化肽活性的保持。通过简单的缓冲梯度调节,纳米微球可以实现对单、多磷酸化肽的选择性富集。此材料不仅可以从标准蛋白质混合物、脱脂牛奶和大鼠脑脊液中选择性富集磷酸化肽,还可以从模型蛋白质混合物、脱脂牛奶和蛋清中特异性捕获磷酸化蛋白质。该研究工作进一步表明,借助多重氢键相互作用,结合聚合物链上丰富的结合位点,可以实现对磷酸化肽的选择性富集。由于该Fe_3_O_4_/PDA/PAMA-Arg纳米复合微球材料特异性不高,因此为了增强材料对目标磷酸化肽富集的特异性,Xiong等^[21]^制备了一种新的亲和材料,在聚甲基丙烯酸缩水甘油酯(PGMA)修饰的Fe_3_O_4_微球上引入胍基,通过简单调节上样溶液成分,可以对全部磷酸化肽或仅对多磷酸化肽具有选择性富集能力。该复合材料吸附容量大(200 mg/g),检测灵敏度高(0.5 fmol),富集回收率高(91.30%),特异性强,有利于低丰度磷酸化肽的检测和鉴定。这种材料可以通过简单的调节溶剂梯度选择性富集单、多磷酸化肽,为开发新型的富集材料提供了新思路。

分子印迹聚合物(MIPs)因其稳定性高、制备简单、可特异性识别、可重复使用等优势被越来越多地用作富集材料。Emgenbroich等^[37]^采用表位印迹法设计了一种针对酪氨酸磷酸化肽的选择性印迹聚合物受体,对酪氨酸磷酸化位点表现出良好的亲和力,明显更易识别酪氨酸磷酸化肽而非丝氨酸磷酸化肽。与抗体进一步对比,印迹聚合物能够捕获酪氨酸磷酸化肽远多于非磷酸化肽和丝氨酸磷酸化肽。Stefan等^[38]^利用尿素单体与模板分子Fmoc-pTyrOEt,以物质的量比为2:1的条件,制备了一种非印迹聚合物(NIP),该聚合物与印迹聚合物完全相同,但省略了模板。该聚合物每个尿素基团都能与磷酸根离子产生氢键,从而与pTyr侧链的结合位点紧密连接,结果显示该聚合物对相对分子质量较低的酪氨酸磷酸化肽表现出很强的亲和力。

在此基础上,Xia和Yang^[22]^采用表位印迹法^[39]^合成了一种温敏性分子印迹聚合物(TMIPs),其制备过程见图2。由于采用了苯基磷酸作为模板,所制备的TMIPs对靶标的酪氨酸磷酸化肽具有较强的识别能力和选择性。当温度为28 ℃时,聚合物空腔尺寸正好匹配苯基磷酸位点,TMIPs对混合肽样品中的酪氨酸磷酸化肽展现出选择性吸附行为,而对非修饰肽段或者丝氨酸、苏氨酸磷酸化肽的吸附能力很弱。当温度提升至28 ℃以上时,聚合物发生明显收缩,导致印迹空腔尺寸显著降低,从而将捕获的酪氨酸磷酸化肽释放出来,便于质谱鉴定。经过4次再结合循环后,材料仍保持对酪氨酸磷酸化肽78.9%的吸附效率。该材料同时兼顾了分子印迹聚合物优异的靶向性,以及温敏型聚合物的温度响应性,为开发其他类型的翻译后修饰富集材料提供了有益的启示。

**图 2:**
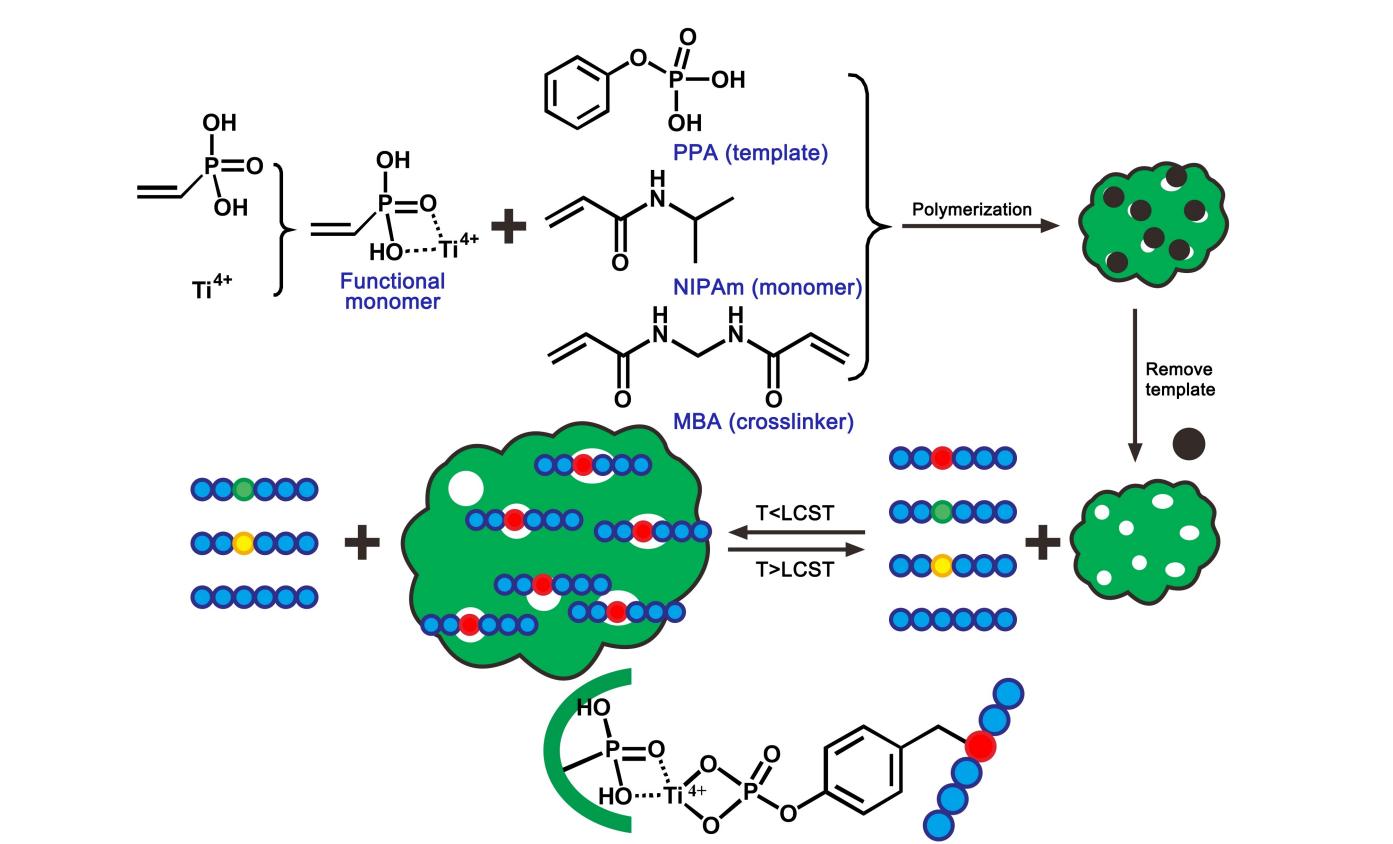
温敏型分子印迹聚合物的合成和工作模式图^[22]^

## 2 智能聚合物基糖肽富集材料

蛋白糖基化与受体激活、信号转导、凝血、免疫反应、细胞死亡和发育等多种生物学活动密切相关,是最复杂、最关键的蛋白质翻译后修饰之一。糖蛋白组学因其在生命科学和医学上的重要意义,受到越来越多的关注^[40]^。充分的证据表明,癌症等重大疾病的发生与蛋白糖基异常相关,目前临床上使用的肿瘤生物标志物大多是糖蛋白^[41]^。传统的糖肽富集材料大部分是基于单分子层的材料,它们与糖肽之间的亲和力受到其接枝密度极大的限制,因此将糖肽富集材料从二维的单层扩展到三维的聚合物将有助于特异性富集能力的大幅提升。

2015年,Qian等^[23]^制备了一种基于智能聚合物的糖肽富集材料。在弱酸性条件下,这种pH敏感型的聚丙烯酸-co-酰肼能够在水中溶解分散,与蛋白质或肽段发生充分接触,均相的作用环境促进了材料对痕量糖肽的捕获(见图3a)。在溶液的pH值降至2.0的条件下,该聚合物迅速自组装成多个大颗粒团聚体,并从溶液中析出,借助快速固液分离,实现了目标糖肽与干扰物的有效脱离。聚合物从均质到非均质状态的快速转化,在1 h内达到95%的转化率,比使用传统固体/不溶性琼脂糖珠的转化率快了8倍(见图3b)。这种pH敏感型的富集材料从小鼠大脑裂解液中共富集出了458个*N*-糖蛋白以及1317个*N*-连接糖肽,进一步验证了该材料在富集实际样品中糖蛋白和糖肽的可行性。此外,与3种主流富集材料相比,聚丙烯酸-酰肼材料对富集到的唾液酸化糖肽表现出更强的信号敏感性(见图3c)。

**图 3:**
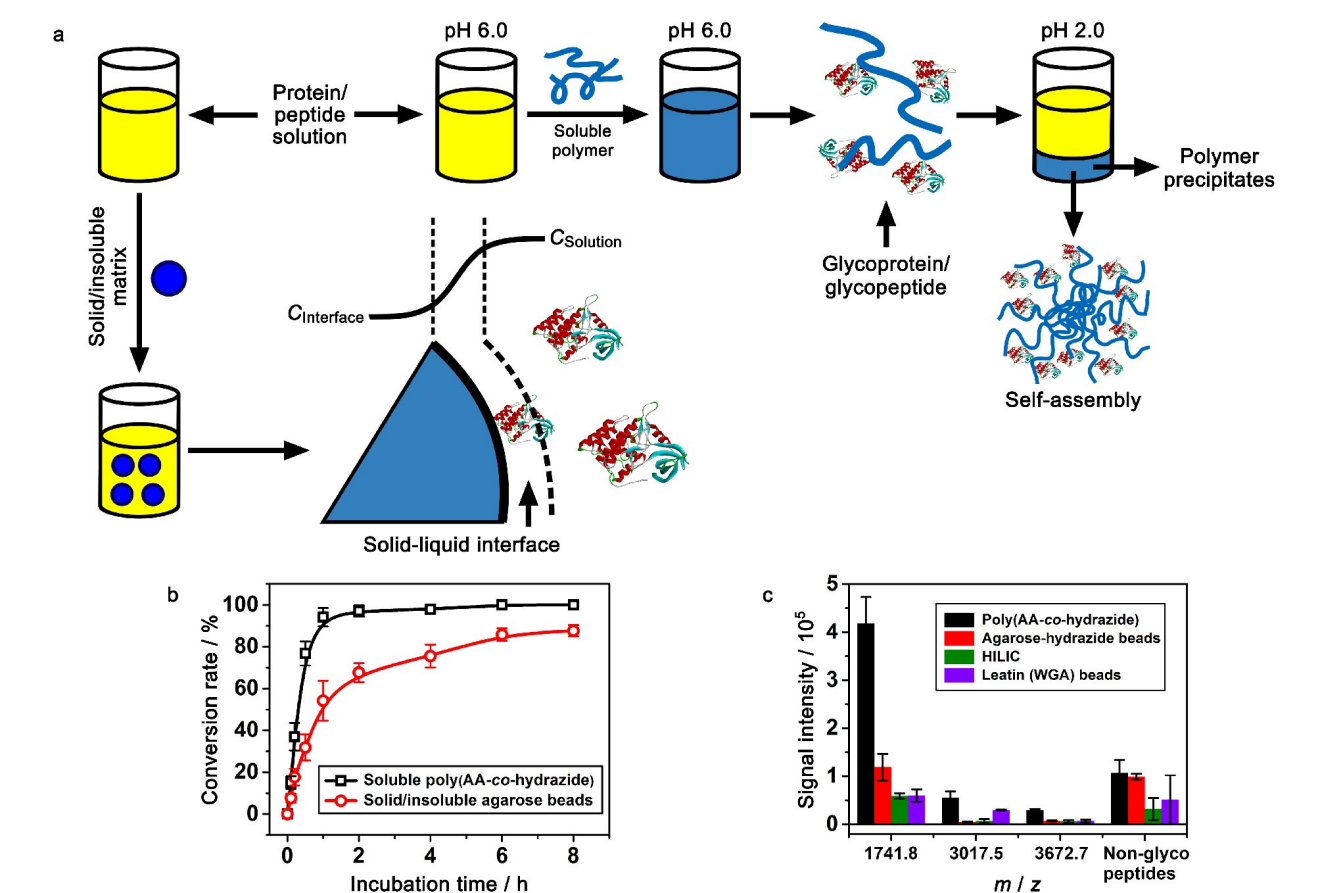
聚丙烯酸-co-酰肼材料与常规非均相材料对糖肽以及糖蛋白富集模式的对比图^[23]^

2017年,Qian等^[24]^还设计了一种聚异丙基丙烯酰胺温敏型聚合物,同时偶联了三苯基膦试剂(见图4a),用于从复杂生物样品中捕获叠氮标记的*O*-GlcNAc糖蛋白。该聚合物在水中溶解性良好,界面传质阻力低,空间位阻小,有利于均相反应的发生。该聚合物通过Staudinger反应促进负载的三苯基膦与叠氮标记的*O*-GlcNAc蛋白之间实现偶联(见图4b)。同时,可以通过调节溶液温度,使得聚合物从溶解状态到完全析出,实现从溶液中快速回收聚合物与*O*-GlcNAc蛋白偶联产物。利用这种新型的固定化三芳基膦试剂,研究人员从HeLa细胞裂解液中通过质谱鉴定到超过1700个潜在的*O*-GlcNAc型糖蛋白。

**图 4:**
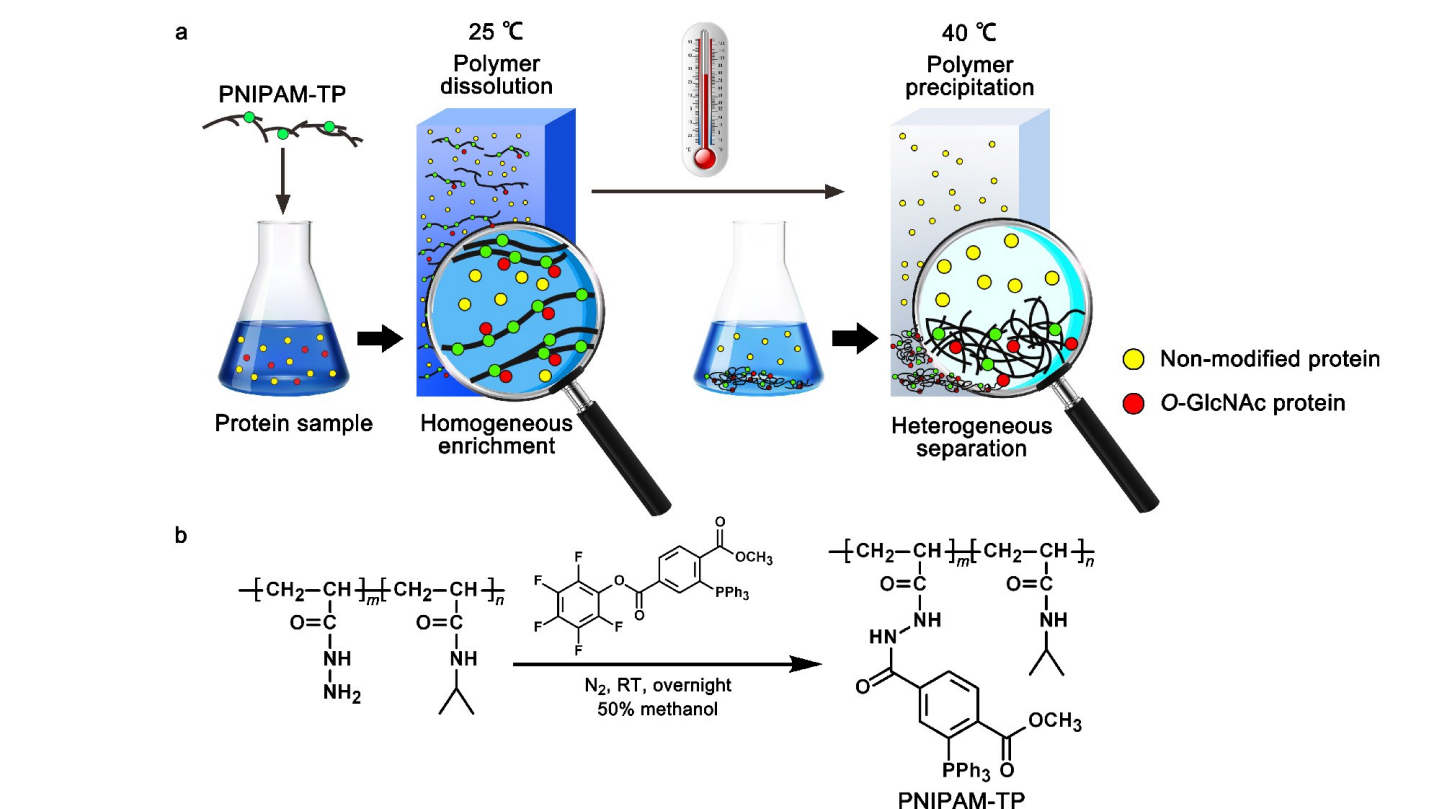
基于可溶性和热敏性PNIPAM-三芳基膦均相富集*O*-GlcNAc蛋白的策略^[24]^

借助苯基硼酸(PBA)与含有顺式二醇化合物之间存在的可逆硼二酯键,PBA材料被广泛用于碱性缓冲液中富集糖肽。2014年,Li等^[41]^研制了一种聚PBA修饰的硅胶材料,基于其选择性和疏水相下的亲水作用,实现了对糖肽的高选择性结合。然而传统的单层PBA材料不可避免地会遇到负载效率低、孵育时间长、选择性差等问题。Gao等^[25]^通过模板共价固定和表面印迹,开发了温敏型分子印迹聚合物微球,用于选择性分离卵清糖蛋白(OB)。首先合成了聚甲基丙烯酸甲酯(PMMA)-co-PBA纳米微球,然后通过PBA与糖可逆的共价键,将模板分子OB固定在微球表面上。随后引发NIPAAm和丙烯酰胺在纳米微球表面的聚合,形成均匀的聚合物涂层。在去除OB模板后,得到分子印迹纳米微球(见图5)。利用PBA与OB结合时的pH依赖性,该材料对OB具有高度可控的吸附、脱附行为和良好的选择性识别能力,被成功地应用于选择性分离来自蛋清样品中的OB。值得指出的是,该印迹纳米球在20 min内达到饱和吸附,表明吸附速率快。

**图 5:**
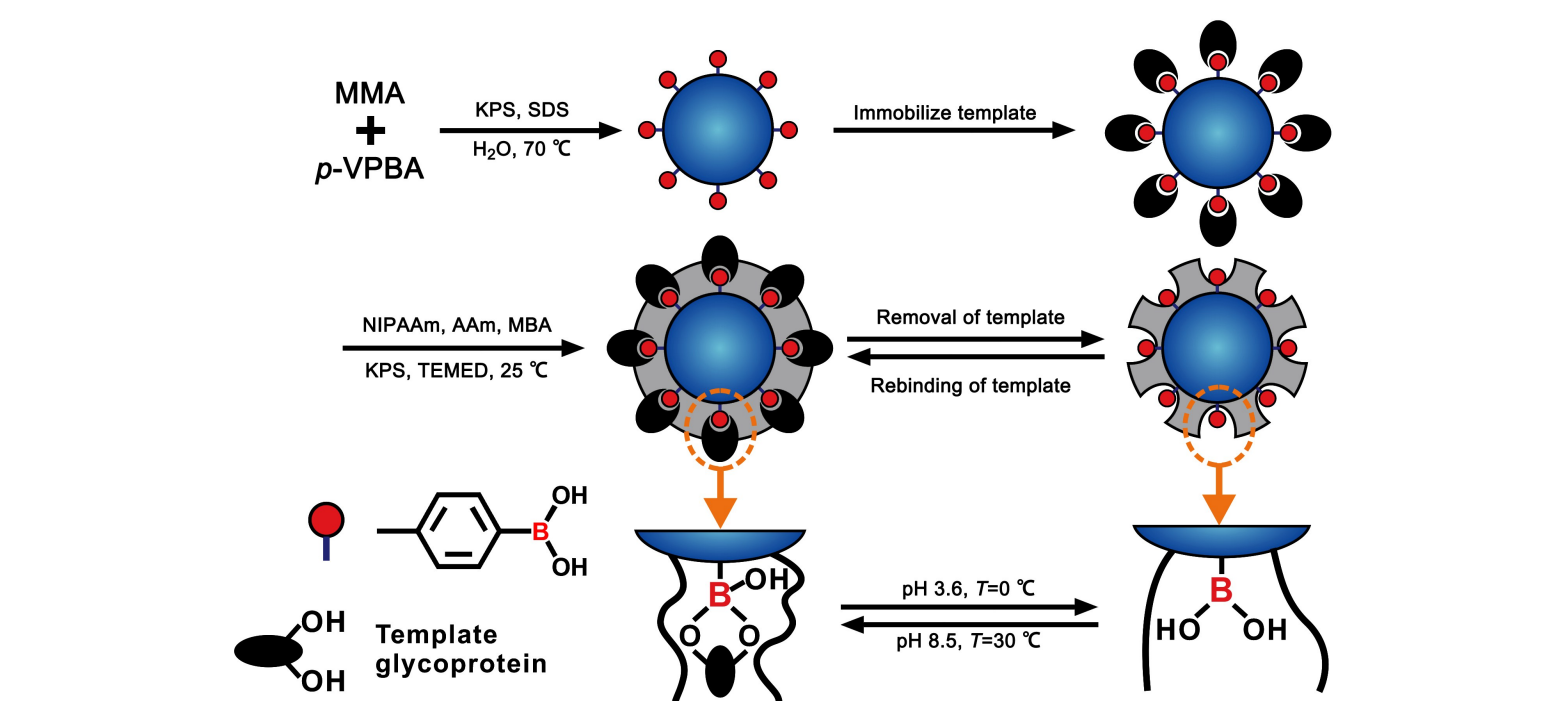
用于选择性分离卵清糖蛋白的温敏型聚合物修饰的分子印迹核壳纳米微球的制备示意图^[25]^

糖肽富集的基础是材料对靶标糖链的特异性捕获和灵敏响应。在糖响应聚合物的构筑方面,Qing和Sun等^[42]^在2011年设计了一种包括识别单元、介导单元和柔性聚合物主链PNIPAM的三组分共聚物聚(*N*-异丙基丙烯酰胺)-甲基酯化-天门冬氨酸-苯丙氨酸二肽-双(三氟甲基)改性苯硫脲(PNIPAM-*β*-MAP-di-TFP)薄膜。初始时,共聚物由于识别单元MAP和介导单元TFP之间的氢键相互作用,使得聚合物链收缩卷曲,薄膜处于超疏水的状态。当聚合物薄膜浸泡在单糖溶液中时,来苏糖分子通过多重氢键作用结合MAP识别单元,导致共聚物氢键网格的破坏,聚合物链发生了从紧缩向完全舒张的转变,伴随着薄膜从超疏水向超亲水的剧烈变化,从而实现了对来苏糖的灵敏响应和检测。采用类似的策略,该研究组设计了一种在聚乙烯基亚胺(PEI)修饰甲基酯化二肽(D-Asp-D-Phe, DF)的智能聚合物基材料薄膜(PEI-g-D-DF)^[43]^。该聚合物膜由L-核糖溶液处理后,变得更加柔软;而与D-核糖溶液作用后,变得更加坚硬,这一手性效应为测定L/D-核糖的对映体纯度,提供了一种全新方法,在此基础上开发出的手性色谱柱实现了对脱氧核糖对映异构体的手性分离,以及对多种单糖、二糖和寡糖的高效分离。这些研究工作表明糖响应型聚合物在糖分离,糖肽富集等领域具有良好的应用前景。

2016年,Qing等^[26]^建立了一种基于亲水指数的二肽序列筛选策略,从54种二肽中筛选出3种最优的二肽序列(见图6a)。优化得到的聚合物材料聚(Pro-Glu)@SiO_2_在糖肽富集方面表现出优异的性能,可以从1:1000倍胎球蛋白与牛血清白蛋白酶解液的混合液中,富集鉴定到33个糖肽信号,对HeLa细胞裂解液中糖肽的富集选择性达到70%。同时,该二肽聚合物材料对不同组成、不同聚合度甚至不同连接异构体的寡糖、糖链均表现出高效的色谱分离。材料将对糖肽的高选择性富集与对糖链结构的精细区分融为一体,代表了新一代糖肽分离材料发展的方向。

**图 6:**
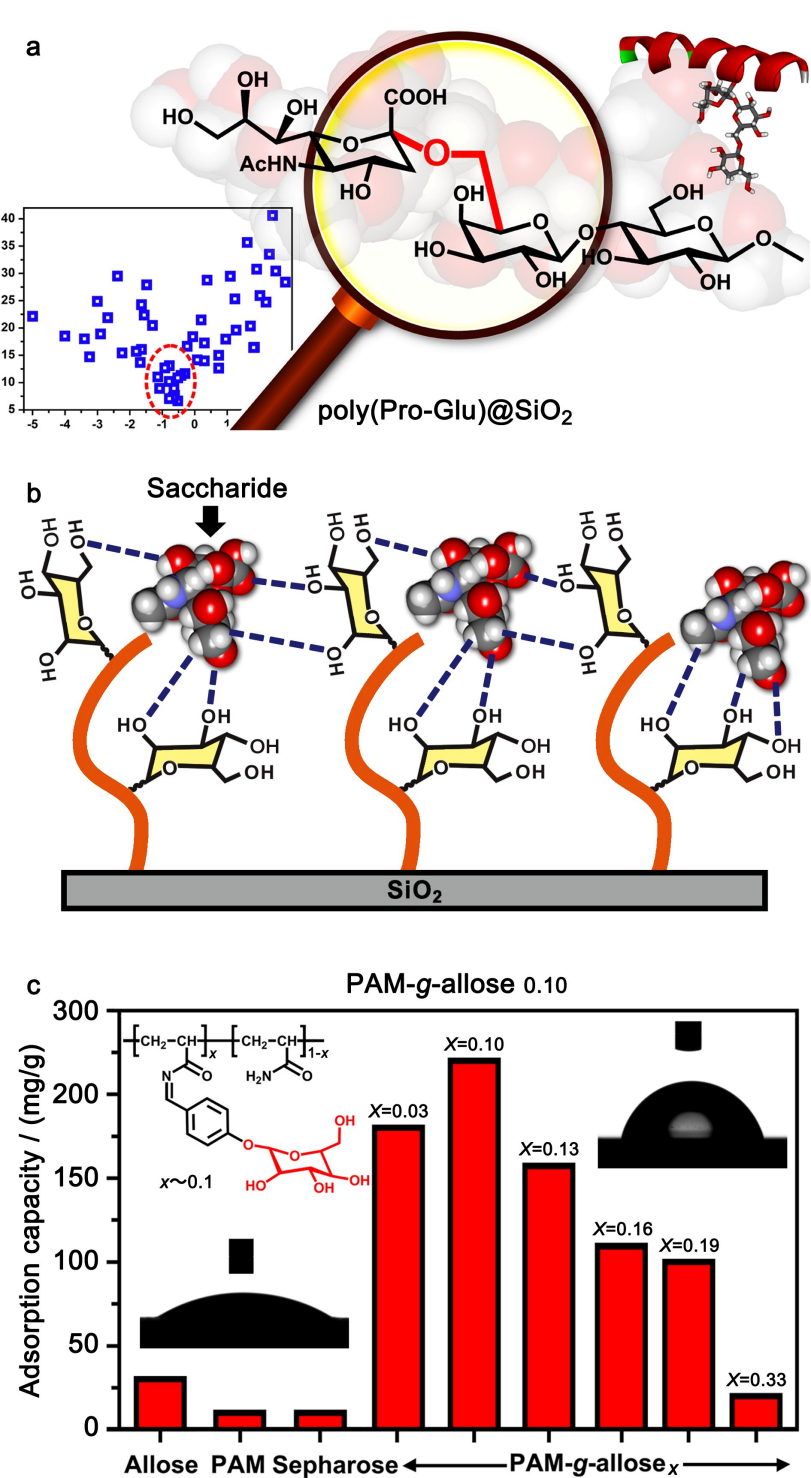
聚合物材料的筛选及其对糖肽的富集^[26,27]^

随后,该研究组受到糖-糖相互作用的生物启发,将阿洛糖引入到聚丙烯酰胺链中,制备了一种糖响应型的聚合物材料^[27]^。在pH为4的弱酸性条件下,阿洛糖识别受体可以选择性地结合唾液酸(见图6b);然而阿洛糖单元在聚合物中的比例至关重要,接枝比例低时,材料对唾液酸糖肽的吸附能力较弱;而接枝比例过高时,聚合物链又会因为链内的氢键相互作用,处于紧缩卷曲的状态。当阿洛糖引到聚丙烯酰胺链的接枝率为0.10时(见图6c),聚合物对唾液酸糖肽表现出最佳的吸附容量(220 mg/g)和较好的富集能力,可以从1:500倍胎球蛋白与牛血清白蛋白酶解液混合液中,富集鉴定到15个糖肽信号,同时从HeLa细胞裂解液中鉴定得到180个唾液酸糖基化位点。此外,该聚合物薄膜体现出明显的疏水性(水接触角大约80°),改变了人们对传统富集材料的认识,它们通常具有很高的亲水性(水接触角大约15°)。基于类似的设计思想,Li等^[44]^还开发了其他基于乳糖的聚合物材料,用于唾液酸糖肽富集。

2017年,Qing等^[45]^将研究从糖链末端的唾液酸扩展至糖链片段,设计的基于L-Asp-L-Phe的二肽能够对唾液酸、*N*-乙酰氨基葡萄糖(GlcNAc)、半乳糖(Gal)和甘露糖(Man)产生强烈且有差异性的结合,正好契合Neu5Ac-Gal-GlcNAc-Man序列的唾液复合体型聚糖。石英晶体微天平吸附实验表明,L-Asp-L-Phe二肽接枝的聚乙烯亚胺薄膜,对模型的唾液酸三糖具有显著的吸附行为,能够清晰地区分它们的连接异构体。这些特性有助于利用PEI-g-L-Asp-L-Phe修饰的mSiO_2_@SiO_2_@Fe_3_O_4_核-壳结构微球,从复杂的蛋白酶解液中选择性捕获到唾液酸糖肽。该研究将有力推动糖链靶向型分离材料的开发,为糖链特异性生物材料的开发指明了方向。Liu等^[28]^用己二酸二肼对Fe_3_O_4_@聚甲基丙烯酸(Fe_3_O_4_@PMAA)表面进行改性,得到一种肼功能化的核-壳结构磁性微球Fe_3_O_4_@PMAH。材料表面丰富的肼基实现了对糖肽的高选择性捕获,Fe_3_O_4_核心优异的磁性便于实现大规模、高通量和自动化的样品处理。此外,亲水性聚合物表面可以降低肽链的非特异性吸附。与商用化肼树脂富集材料相比,Fe_3_O_4_@PMAH的富集得到糖肽的信噪比提高了5倍以上。该纳米材料在对结直肠癌患者血清*N*-连接糖蛋白的表征中,鉴定出175条糖肽和181个糖基化位点,以及63个不同的糖蛋白位点,富集糖肽和相应糖蛋白的选择性分别为69.6%和80.9%。

多孔聚合物材料同样也被广泛应用于生物分离,然而传统的均质多孔聚合物材料不能有效地从复杂样品中分离出特定的低丰度生物分子。2015年,Jin等^[46]^提出了一种简便的无模板合成中孔酚醛聚合物的方法,合成的聚合物具有较高的BET比表面积(548 m^2^/g)和中孔尺寸(13 nm),并显示出优异的糖肽类捕获性能。用nano LC-MS/MS进行3次平行实验后,能够成功地鉴定出169个独特的糖肽和92个糖蛋白,展现了中孔聚合物在高选择性糖肽富集方面的潜在应用。2018年,Song等^[47]^报道了通过乳液界面聚合法制备具有大小可调的异质结构纳米孔的颗粒,用于实现复杂生物样品中低丰度糖肽的分离。纳米孔内的异质结构表面,允许生物分子依赖于溶剂的局部吸附到亲水或疏水区域;在疏水区域去除高丰度疏水蛋白和非修饰肽后,通过低极性溶剂中纳米孔的亲水区域,可以有效地富集到低丰度亲水型糖肽。这些具有异质结构纳米孔的颗粒有望用于核酸、糖类和蛋白质的分离及下游的临床诊断。

## 3 结论

智能聚合物基材料能够动态响应外界的各种物理和化学刺激,发生剧烈的构象变化,从而引起表界面物理化学性质的大幅转变,这为生物分离分析提供了一个额外的驱动力,能够大幅提升生物分离分析方法的灵敏性、选择性和响应速率。基于翻译后修饰蛋白组的富集材料的开发,智能聚合物基材料,特别是生物分子敏感型聚合物,能够有效解决材料富集选择性不足、吸附效率低、难以洗脱等难题,有望实现更广泛的应用,是翻译后修饰蛋白富集和蛋白前处理的理想材料。

然而一些难题和挑战有待解决:第一,目前智能聚合物基材料大多面向模型蛋白体系,包括混合有牛血清白蛋白的复杂样品、细胞裂解液,甚至血液等,这些样品与人体实际样本在复杂性上还有很大的差异性。智能聚合物基材料在复杂人体环境中的工作能力还有待观察和体验,这需要分析化学家与医学临床科研人员更加紧密地合作,让分析方法更加贴近实际需求。第二,分离分析方法对数据的重现性提出了很高要求,对于同一样本的不同批次处理,数据集之间应具有很高的重叠性。为此智能聚合物基材料需要着力提升材料的可逆性,实现材料高质量的可重复利用,为测量数据的可重复性提供有力保障。第三,智能材料应大力扩展应用范围,将研究重心从磷酸化肽、唾液酸糖肽,逐渐扩展至其他翻译后修饰类型,例如*O*-连接型糖肽、中性糖糖肽、甲基化肽、乙酰化肽、泛素化肽等,可以预测智能聚合物基材料将在翻译后修饰蛋白质组学中获得更为广阔和深入的应用。
